# Shenxiong glucose injection inhibits oxidative stress and apoptosis to ameliorate isoproterenol-induced myocardial ischemia in rats and improve the function of HUVECs exposed to CoCl_2_

**DOI:** 10.3389/fphar.2022.931811

**Published:** 2023-01-05

**Authors:** Zhong-Xiu Wu, Shuai-Shuai Chen, Ding-Yan Lu, Wei-Na Xue, Jia Sun, Lin Zheng, Yong-Lin Wang, Chun Li, Yong-Jun Li, Ting Liu

**Affiliations:** ^1^ State Key Laboratory of Functions and Applications of Medicinal Plants and Guizhou Provincial Key Laboratory of Pharmaceutics, Guizhou Medical University, Guiyang, China; ^2^ School of Pharmacy, Guizhou Medical University, Guiyang, China; ^3^ Engineering Research Center for the Development and Application of Ethnic Medicine and TCM (Ministry of Education) and State Key Laboratory of Functions and Applications of Medicinal Plants, Guizhou Medical University, Guiyang, China; ^4^ School of Medicine and Health Management, Guizhou Medical University, Guiyang, China

**Keywords:** Shenxiong glucose injection, myocardial ischemia, reactive oxygen species, oxidative stress, apoptosis, HUVECs

## Abstract

**Background:** Shenxiong Glucose Injection (SGI) is a traditional Chinese medicine formula composed of ligustrazine hydrochloride and Danshen (Radix et rhizoma *Salviae miltiorrhizae*; *Salvia miltiorrhiza* Bunge, Lamiaceae). Our previous studies and others have shown that SGI has excellent therapeutic effects on myocardial ischemia (MI). However, the potential mechanisms of action have yet to be elucidated. This study aimed to explore the molecular mechanism of SGI in MI treatment.

**Methods:** Sprague-Dawley rats were treated with isoproterenol (ISO) to establish the MI model. Electrocardiograms, hemodynamic parameters, echocardiograms, reactive oxygen species (ROS) levels, and serum concentrations of cardiac troponin I (cTnI) and cardiac troponin T (cTnT) were analyzed to explore the protective effect of SGI on MI. In addition, a model of oxidative damage and apoptosis in human umbilical vein endothelial cells (HUVECs) was established using CoCl_2_. Cell viability, Ca^2+^ concentration, mitochondrial membrane potential (MMP), apoptosis, intracellular ROS, and cell cycle parameters were detected in the HUVEC model. The expression of apoptosis-related proteins (Bcl-2, Caspase-3, PARP, cytoplasmic and mitochondrial Cyt-c and Bax, and p-ERK1/2) was determined by western blotting, and the expression of cleaved caspase-3 was analyzed by immunofluorescence.

**Results:** SGI significantly reduced ROS production and serum concentrations of cTnI and cTnT, reversed ST-segment elevation, and attenuated the deterioration of left ventricular function in ISO-induced MI rats. *In vitro*, SGI treatment significantly inhibited intracellular ROS overexpression, Ca^2+^ influx, MMP disruption, and G2/M arrest in the cell cycle. Additionally, SGI treatment markedly upregulated the expression of anti-apoptotic protein Bcl-2 and downregulated the expression of pro-apoptotic proteins p-ERK1/2, mitochondrial Bax, cytoplasmic Cyt-c, cleaved caspase-3, and PARP.

**Conclusion:** SGI could improve MI by inhibiting the oxidative stress and apoptosis signaling pathways. These findings provide evidence to explain the pharmacological action and underlying molecular mechanisms of SGI in the treatment of MI.

## 1 Introduction

Ischemic heart disease accounts for approximately 50% of all cardiovascular diseases (CVDs) and is the leading cause of human mortality (Fan et al., 2017). Myocardial ischemia (MI) is defined pathologically as myocardial cell death due to prolonged ischemia (Thygesen et al., 2018) and is a major challenge in the clinical setting (Wu et al., 2019; Zhai K. et al., 2021). The development of MI is marked by complex molecular mechanisms, such as Ca^2+^ overload (Chang et al., 2019), reactive oxygen species (ROS) accumulation, and apoptosis (Bugger and Pfeil, 2020), and these mechanisms are driving the search for new drugs and therapies for the prevention and treatment of MI.

**FIGURE 1:**
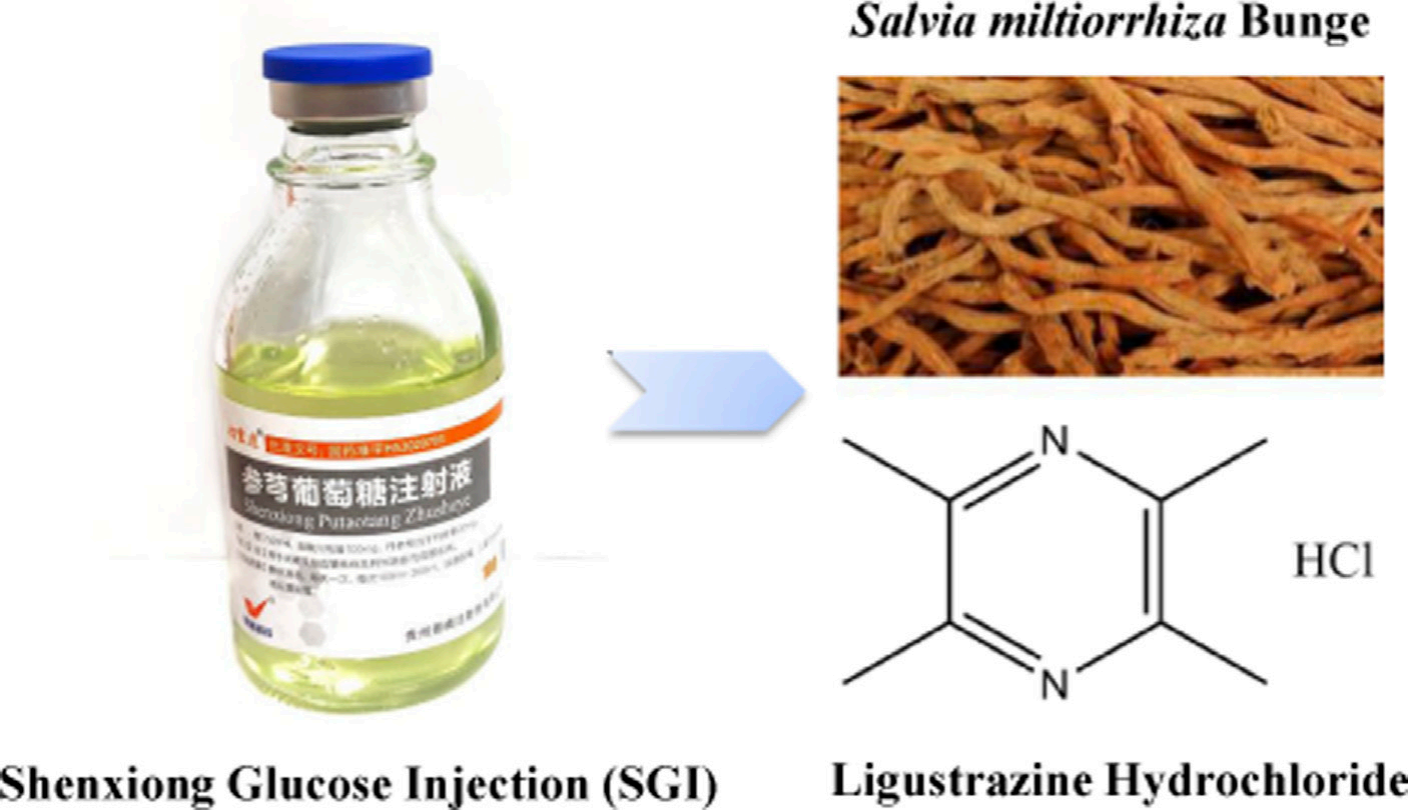
SGI and its compositions.

Traditional Chinese medicine (TCM) and its formulas are the most common therapy for CVD in China because of their superiority and wide-ranging regulatory effects (Wang et al., 2018). Examples of TCM for the treatment of CVD include Shenxiong Glucose Injection (SGI), Danhong injection (Zhai S. et al., 2021), and Shensong Yangxin Capsule (Jiang et al., 2021). SGI (Figure 1) is a TCM injection, composed of ligustrazine hydrochloride and Danshen (Radix et rhizoma *Salviae miltiorrhizae*; *Salvia miltiorrhiza* Bunge, Lamiaceae). It has the function of activating blood circulation to dissipate blood stasis (Zheng, 2015). In addition, the pharmacological functions of SGI include microcirculation improvement, thrombosis inhibition, antiendothelial injury, antioxidation, anti-inflammatory, and antiplatelet aggregation effects (Zhang J. et al., 2022). SGI is widely used in the clinical setting for the treatment of various disease including MI, coronary heart disease, acute ischemic stroke, and angina pectoris (Zheng, 2015; Zhou et al., 2018). Randomized controlled trials demonstrated that SGI significantly improves total efficiency, exiting rate, and health quality in CVD (Sun et al., 2017; Lv et al., 2019). Moreover, SGI has a significant curative effect on central system diseases, such as cerebral ischemia, acute cerebral infarction, vertebrobasilar insufficiency vertigo, etc. (Li et al., 2017). However, there are limited studies exploring the underlying functional mechanism(s) of SGI.

Apoptosis is a highly regulated process of cell death (Xu et al., 2019). Human vascular endothelial cell apoptosis is closely related to the occurrence and development of various CVDs related to high mortality (Zhai et al., 2017; Gong et al., 2019). Hypoxia induction is an important factor in the pathophysiological mechanism of MI (Cheng et al., 2016). Oxidative stress leads to apoptosis of vascular endothelial cells, which can respond immediately to changes in hypoxic stimulation owing to their direct contact with blood (Yang et al., 2018). Inhibiting the apoptosis of vascular endothelial cells is considered an important means of restoring blood and nutrient supply to the damaged area and thus improving CVD (Kukumberg et al., 2021). Human vascular endothelial cell dysfunction and apoptosis are critical for the occurrence and development of CVD (Zhai et al., 2015; Wu et al., 2019). Human umbilical vein endothelial cells (HUVECs) are a standard model for studying human vascular endothelial cells at the cellular level (Sun et al., 2021).

In this study, the pharmacological action and molecular mechanisms of SGI in the treatment of MI were investigated in a rat model of MI induced by isoproterenol (ISO) and in HUVECs with oxidative damage and apoptosis induced by CoCl_2_. Findings from the study provide a scientific basis for future research on SGI application in the treatment of MI.

## 2 Materials and methods

### 2.1 Materials and reagents

SGI (batch no. 07210312; date of production: 20210319; standard no: WS-10001-(HD-1136)-2002-2012-2017) was obtained from Guizhou Jingfeng Injection Co., Ltd. (Guiyang, China). The ingredients of the SGI are (per 100 mL): 100 mg ligustrazine hydrochloride, Danshen (equivalent to 20 mg danshensu), 5.0 g glucose, 1.0 mL glycerin). The chemical profile of SGI refers to our previous studies Supplementary Figure S1 (Zheng, 2015). ISO and CoCl_2_ were purchased from Sigma-Aldrich (MO, United States). The PI/RNase Staining Buffer and the Annexin V-FITC/PI apoptosis detection kit were purchased from BD Biosciences (CA, United States). ROS, Ca^2+^, mitochondrial membrane potential (MMP), cell mitochondria isolation kits, and bicinchoninic acid (BCA) kits were acquired from Beyotime Biotechnology (Shanghai, China). ROS detection kit (Nanjing Jiancheng Bioengineering Institute, Nanjing, China). Antibodies against extracellular signal-related kinases1/2 (ERK1/2), poly-ADP ribose polymerase (PARP), and phosphorylation extracellular signal-related kinases1/2 (p-ERK1/2) were purchased from Cell Signaling Technology (MA, United States), while an antibody against cytochrome c oxidase IV (COX-IV) was purchased from Proteintech Group, Inc., (Wuhan, China). B cell lymphoma-2 (Bcl-2), cysteinyl aspartate specific proteinase 3 (Caspase-3), Bcl-2-associated X protein (Bax), and cleaved caspase-3 were purchased from Abcam (Cambridge, United Kingdom). Glyceraldehyde-3-phosphate dehydrogenase (GAPDH) was purchased from Thermo Fisher Scientific (MA, United States). CellTiter 96® AQueous One Solution Cell Proliferation Assay kit was purchased from Promega (WI, United States). Bovine serum albumin (BSA), phosphate buffer saline (PBS), radio immunoprecipitation assay (RIPA) lysis buffer, and phenylmethanesulfonyl fluoride (PMSF) were obtained from Solarbio Technology Co., Ltd., (Beijing, China). Polyvinylidene fluoride (PVDF) membranes were purchased from Merck Millipore (MA, United States). Normal saline (NS) and glucose injection (GS) were acquired from Guizhou Kelun Pharmaceutical Co., Ltd., (Guiyang, China). RPMI-1640 medium and fetal bovine serum were purchased from Gibco (NY, United States). Serum cardiac troponin T (cTnT) and cardiac troponin I (cTnI) assay kits were purchased from Shanghai Zhuocai Biotechnology Co., Ltd., (Shanghai, China).

### 2.2 Animals and treatments

**FIGURE 2:**
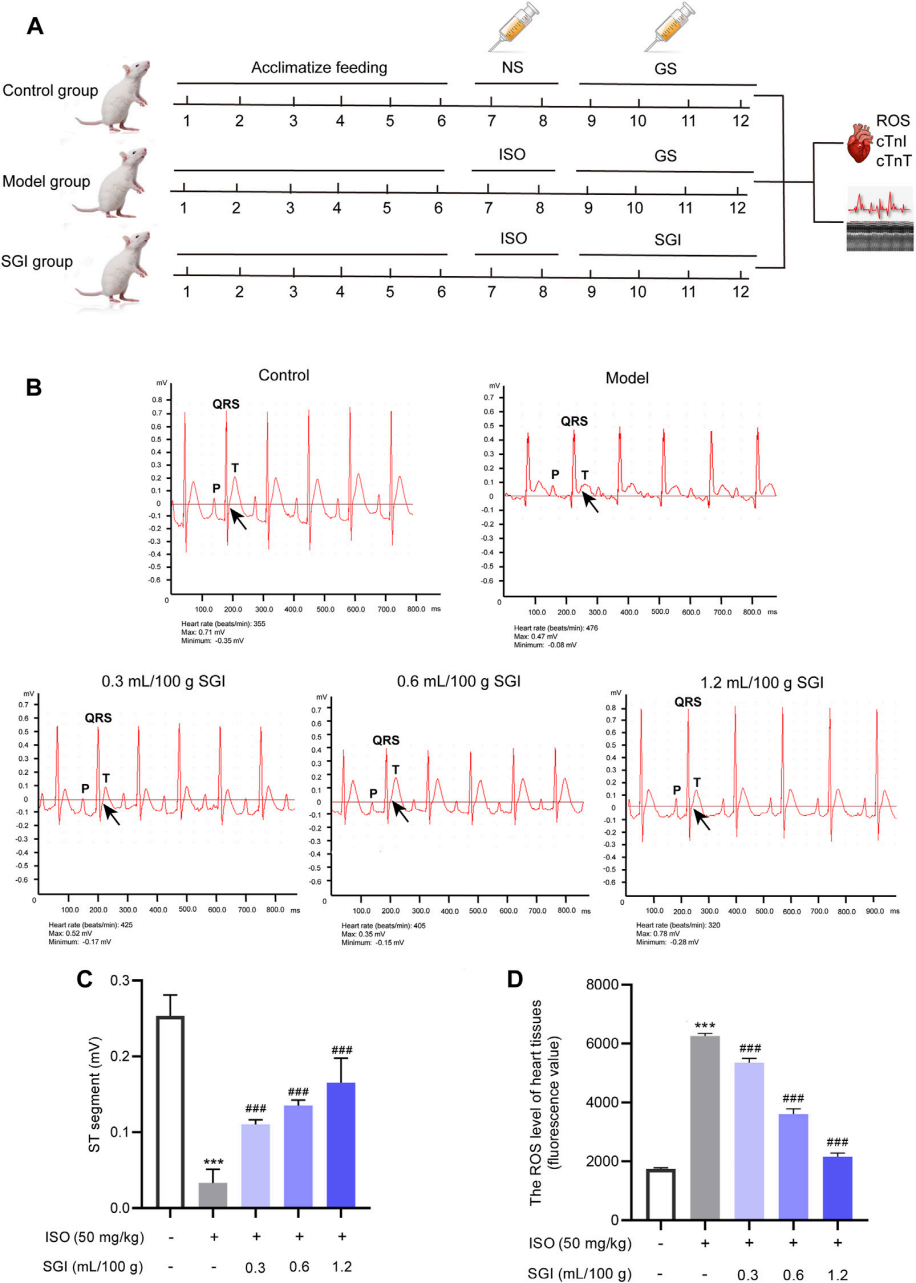
SGI improves ST-segment elevation and decreases ROS. **(A)** Study design of the current study. Detection of the ECG **(B)**, ST-segment values **(C)**, and the ROS level in the heart tissues (fluorescence value) **(D)** of MI rats by using a BL-420 biological function experiment system and DCFH-DA probe, respectively. ^***^ *p* < .001 vs. the control group; ^###^ *p* < .001 vs. the model group.

Specific pathogen-free (SPF) male Sprague-Dawley rats were obtained from the Guizhou Medical University Laboratory Animal Center (permission no. SCXK (Qian) 2018-0001). All rats (weight 200–220 g, 7–8-week-old) were randomly divided into five experimental groups (*n* = 6 per group): control, model, and low-dose, medium-dose, and high-dose SGI. The rats were injected subcutaneously with ISO (50 mg/kg/day) for 2 days to induce a MI model. The dose and pattern of injection were conducted according to a previous study (Sammeturi et al., 2019). Subsequently, the control and model groups were injected with GS (.6 ml/100 g, iv.), while the SGI low-, medium-, and high-dose SGI groups were given SGI (.3, .6, and 1.2 ml/100 g, iv, respectively; equivalent to 1/6, 1/3, and 2/3 of the clinical doses; equivalent to 3 mg/kg ligustrazine hydrochloride and .6 mg/kg danshensu, 6 mg/kg ligustrazine hydrochloride and 1.2 mg/kg danshensu, and 12 mg/kg ligustrazine hydrochloride and 2.4 mg/kg danshensu) for 4 days. All rats were sacrificed 24 h after the final treatment. The study design is shown in Figure 2A. The animal experiments were reviewed and approved by the Animal Care Welfare Committee of Guizhou Medical University.

### 2.3 Measurement of electrocardiogram (ECG)

A standard limb lead II ECG was recorded by a BL-420F biological function experiment system (Chengdu Taimeng Technology Co., Ltd., Chengdu, China) in each group. Four hypodermic needle electrodes were used to record the ECG in anesthetized rats: 1) the left upper limb was connected by a yellow electrode; 2) the left lower limb was connected by a green electrode; 3) the right upper limb was connected by a red electrode; 4) the right lower limb was connected by a black electrode (Song, 2021).

### 2.4 Echocardiographic and hemodynamic assessment of cardiac function

Echocardiograms were performed using a M6Vet ultrasound machine (Shenzhen Mindray Bio-Medical Electronics Co., Ltd., Guangdong, China) equipped with a C11-3S scanning probe transducer. The M-mode recording method was used to measure the left ventricular (LV) end-systolic diameter (LVESD) and LV end-diastolic diameter (LVEDD). Mitral valve E wave peak velocity (MV E vel) was also measured (Qian et al., 2018).

### 2.5 Determination of serum concentrations of cTnI and cTnT

Blood samples of all rats were collected from the femoral artery and centrifuged at 1,000 g for 20 min to isolate serum (Liu et al., 2021). Subsequently, the serum concentrations of the marker enzymes cTnT and cTnI were measured by enzyme linked immunosorbent assay (ELISA) kits (Shanghai Zhuocai Biotechnology Co., Ltd, Shanghai, China) according to the manufacturer’s protocols.

### 2.6 Assay of ROS in rat hearts

As previously described (Hu et al., 2021), heart samples were washed in cold PBS and single-cell suspensions were prepared using mechanical methods. ROS production was determined using a ROS detection kit (Nanjing Jiancheng Bioengineering Institute, Nanjing, China), according to the manufacturer’s instructions. Briefly, cells were collected and washed twice with PBS. Each sample was mixed with 1 mL PBS and 1 μL 2ʹ,7ʹ-dichlorodihydrofluorescein diacetate (DCFH-DA) (final concentration 10 μmol/L) for 30 min at 37°C. ROS fluorescence intensity was measured by using a fluorescence microplate reader (Thermo Scientific, MA, United States).

### 2.7 Cell culture

HUVECs were obtained from the Chinese Academy of Sciences Cell Bank (Shanghai, China) and were cultured in RPMI-1640 medium containing 10% fetal bovine serum at 5% CO_2_ and 37°C. Cells from passages 5–10 were utilized in for the experiments.

### 2.8 Establishment of the HUVECs model and selection of SGI dosage

Cells (8×10^4^ cells/ml) were seeded in 96-well plates and cultured for 24 h. For the establishment of the model, HUVECs in the logarithmic phase were treated with different concentrations of CoCl_2_ (.2–1.4 mmol/L) for 6, 12, 24, and 48 h. For the selection of SGI dosage, HUVECs were treated with different concentrations of GS and SGI (4%–24%, v/v) for 24 h. Cell viability was detected with an MTS (Promega, WI, United States) assay according to the manufacturer’s instructions.

### 2.9 SGI treatment

Cells (8×10^4^ cells/ml) were seeded in 96-well plates and cultured for 24 h. The control group was treated with 2% GS and the CoCl_2_ group was treated with 2% GS and 1.4 mmol/L CoCl_2_. Pretreating with SGI for 6 h, SGI groups were treated with different concentrations of SGI (.5%, 1%, and 2%) combined with 1.4 mmol/L CoCl_2_ for 24 h. Cell viability was detected with an MTS (Promega, WI, United States) assay.

### 2.10 Measurement of intracellular ROS

HUVECs (8×10^4^ cells/ml) were seeded in 6-well plates for 24 h and treated as required. Detection of intracellular ROS was performed as previously described (Long et al., 2022). Briefly, according to the manufacturer’s instructions, HUVECs were stained with DCFH-DA (10 μmol/L) and incubated in the dark for 30 min, and then were washed three times with PBS. The level of intracellular ROS was detected by a fluorescence microplate reader (Thermo Scientific, MA, United States) and fluorescence images of ROS were captured by a confocal laser scanning microscope (CLSM) (Carl Zeiss AG, BW, Germany).

### 2.11 Measurement of cytosolic Ca^2+^, MMP, cell cycle, and apoptosis

HUVECs (8×10^4^ cells/ml) were seeded in 6-well plates for 24 h and treated as required. Cytosolic Ca^2+^, MMP, cell cycle parameters, and apoptosis were respectively detected by the Fluo-4 AM probe (Beyotime Biotechnology, Shanghai, China), the JC-1 probe (Beyotime Biotechnology, Shanghai, China), PI/RNase Staining Buffer (BD Biosciences, CA, United States), and Annexin V-FITC/PI apoptosis detection kit (BD Biosciences, CA, United States), according to the manufacturers’ instructions, using a flow cytometer (BD C6 Plus, BD Biosciences, CA, United States). The data were analyzed using FlowJo 10.6 software (BD Biosciences, CA, United States). A CLSM (Carl Zeiss AG, BW, Germany) was used to capture the fluorescence images of MMP (Qin et al., 2021; Yu et al., 2021; Wu et al., 2022).

### 2.12 Western blot analysis

HUVECs (8×10^4^ cells/ml) were seeded in 6-well plates for 24 h and treated as required. After treatment, HUVECs were lysed with RIPA lysis buffer (containing 1% PMSF). Insoluble materials were removed by centrifugation for 10 min at 12,000 g and 4°C to obtain total protein. The protein concentration was measured by a BCA kit. Equal amounts of protein from each sample were separated by sodium dodecyl sulfate-polyacrylamide gel electrophoresis (SDS-PAGE) and transferred onto a PVDF membrane. Nonspecific sites were blocked by incubating the membranes in 5% BSA buffer. Thereafter, the membranes were incubated with appropriate primary antibodies (GAPDH, 1 in 2000 in BSA buffer; ERK1/2, p-ERK1/2, Bcl-2, Bax, Cyt-c, COX-IV, Caspase-3, and PARP, 1 in 1,000 in BSA buffer; cleaved caspase-3 1 in 500 in BSA buffer) overnight at 4°C. The membranes were washed with tris-buffered saline containing Tween 20 (TBS-T) and incubated with the appropriate secondary antibodies (1 in 2000 in TBS-T buffer). After washing with TBS-T five times (5 min each), the membranes were visualized by a Bio-Rad imaging system (Bio-Rad, CA, United States) (Chen et al., 2022). Analogously, the expressions of Bax and Cyt-c in mitochondria and cytoplasm were measured.

### 2.13 Immunofluorescent analysis

HUVECs (8×10^4^ cells/ml) were seeded in petri dishes for CLSM for 24 h and treated as required. HUVECs were then washed three times with PBS, fixed in 4% paraformaldehyde for 20 min, and incubated in .5% Triton X-100 for 20 min. Subsequently, cells were blocked with 5% BSA at room temperature for 30 min and incubated with cleaved caspase-3 antibody (1:500 in 1% BSA) at 4°C overnight. Subsequently, cells were washed with PBS and incubated with the fluorescent secondary antibody (1:500) for 1 h in the dark at room temperature. The samples were incubated with DAPI (4ʹ,6-diamidino-2-phenylindole) for 5 min and viewed using a CLSM.

### 2.14 Data analysis and statistics

All data are expressed as mean ± standard deviation (SD). SPSS software (IBM, IL, United States) was Student’s *t*-tests were employed to compare the difference between GS and SGI groups. One-way analysis of variance (ANOVA) with a *post hoc* analysis followed by the Least Significant Difference test (for normal distributions) or Dunnett’s *t*-test (for non-normal distributions) was used to compare the difference among multiple groups. *p* < .05 was defined as statistically significant.

## 3 Results

### 3.1 Effect of SGI on ISO-induced MI in rats

ST-segment elevation on an ECG is a crucial indicator to evaluate the degree of MI (Liu et al., 2022). ISO causes MI predominantly through oxidative stress, the important indicator of which is ROS (Wang et al., 2019; Xue et al., 2021). In this study, compared with the control group, the ST-segment of the ECG (black arrow, Figure 2B) was significantly elevated in the ISO-induced rats (Figure 2C), which is constituent with previous literature (Xue et al., 2021). In addition, as shown in Figure 2D, ROS was overproduced in the model group compared with the control group (*p* < .001). However, the three dose groups of SGI exhibited significantly improved ISO-induced ST-segment elevation and ROS production (*p* < .001, Figures 2C, D). These results suggest that SGI produces an obvious antioxidant effect in the treatment of MI.

### 3.2 SGI improves ISO-induced changes in hemodynamic parameters and cardiac function

**FIGURE 3:**
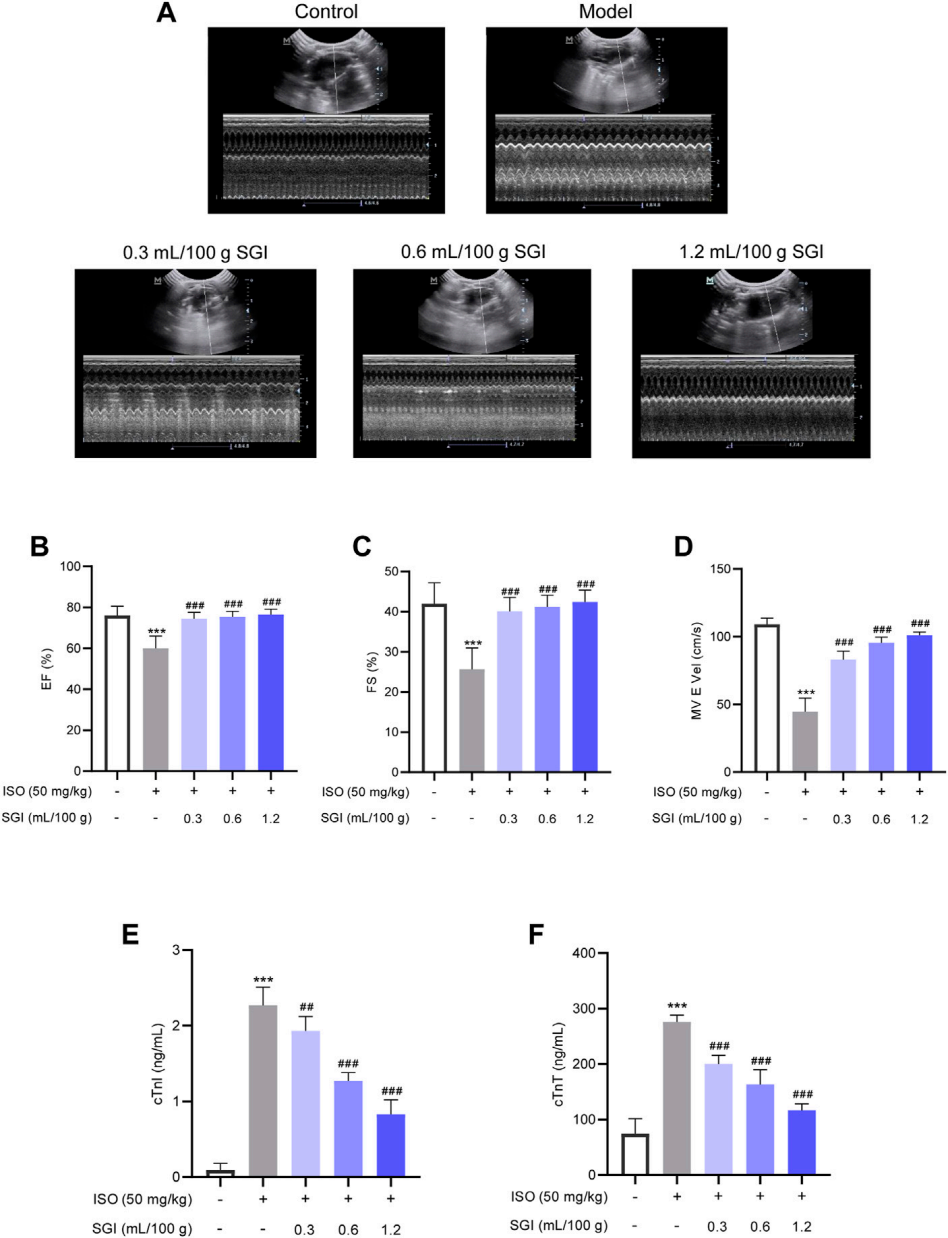
SGI improves ISO-induced changes in cardiac function. **(A)** Representative M-mode echocardiography images. **(B)** Left ventricular ejection fraction [EF(%)] and **(C)** left ventricular fractional shortening [FS(%)] reflect LV systolic function. **(D)** MV E vel reflects LV diastolic function. The effect of SGI on the serum cardiac markers (cTnT **(E)** and cTnI **(F)**) in ISO-induced MI in rats. ^***^ *p* < .001 vs. the control group; ^###^ *p* < .001 vs. the model group.

Echocardiography analysis demonstrated that SGI treatment significantly improved the LV function of MI rats compared with the model group, as evidenced by increasing EF(%) and FS(%) (*p* < .001, Figures 3A, C). The MV E vel is used to evaluate LV diastolic function. SGI treatment (.3, .6, and 1.2 ml/100 g) evaluated ISO-induced MV E vel (*p* < .001, Figure 3D). Furthermore, SGI treatment significantly reduced the serum concentration of cTnT and cTnI (*p* < .001, Figures 3E, F). These results demonstrated that SGI could protected LV systolic and diastolic function against MI.

### 3.3 SGI impairs CoCl_2_-induced injury in HUVECs

**FIGURE 4:**
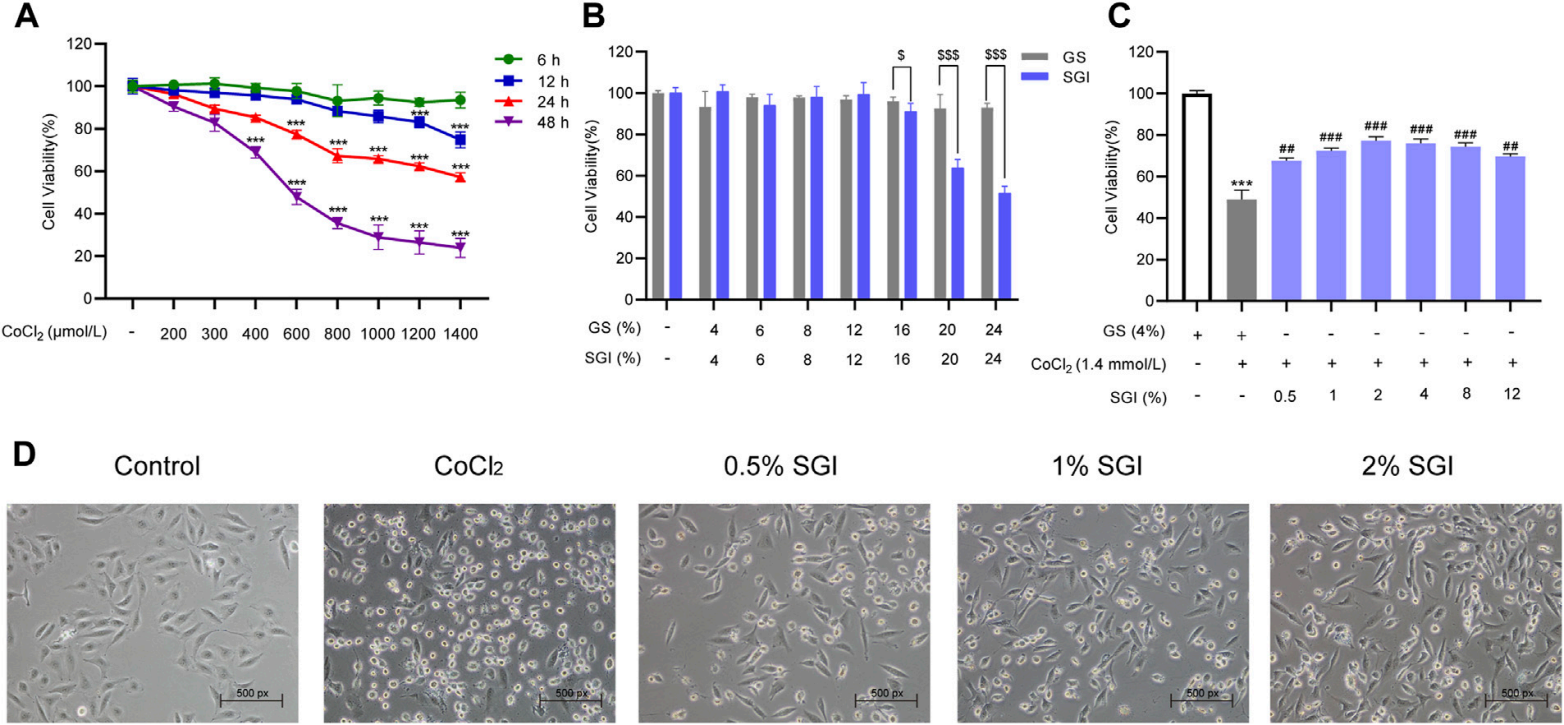
SGI inhibits CoCl_2_-induced injury in HUVECs. **(A)** HUVECs were treated with different concentrations of CoCl_2_ for the indicated times and cell viability was measured by MTS assay. **(B)** HUVECs were treated with SGI and GS from 4% to 24% for 24 h and cell viability **(C)** was measured by MTS assay. **(D)** Cell morphology was examined by a light microscope. ^***^ *p* < .001 vs. the control group; ^##^ *p* < .01, ^###^ *p* < .001 vs. the CoCl_2_ group; ^$^ *p* < .05, ^$$$^ *p* < .001 vs. the GS group.

As CoCl_2_ at 1.4 mmol/L resulted in a 50% decrease in cell survival rate, this was the concentration selected for the establishment of the HUVECs model (Figure 4A). As shown in Figure 4B, there was no difference in the survival rate of HUVECs between the SGI groups and the GS groups at the concentration range of 4%–12% (v/v). However, SGI at the concentration of .5%, 1%, and 2% significantly improved the cell survival rate compared with the CoCl_2_ group (*p* < .01, Figure 4C). Therefore, SGI (concentrations of .5%, 1%, and 2%) were employed for further study. Meanwhile, morphological observations revealed that SGI treatment could reduce the shrinkage and debris of the cells and cell damage induced by CoCl_2_ (Figure 4D). These findings suggest that SGI could impair CoCl_2_-induced injury in HUVECs.

### 3.4 SGI reduces intracellular ROS production

**FIGURE 5:**
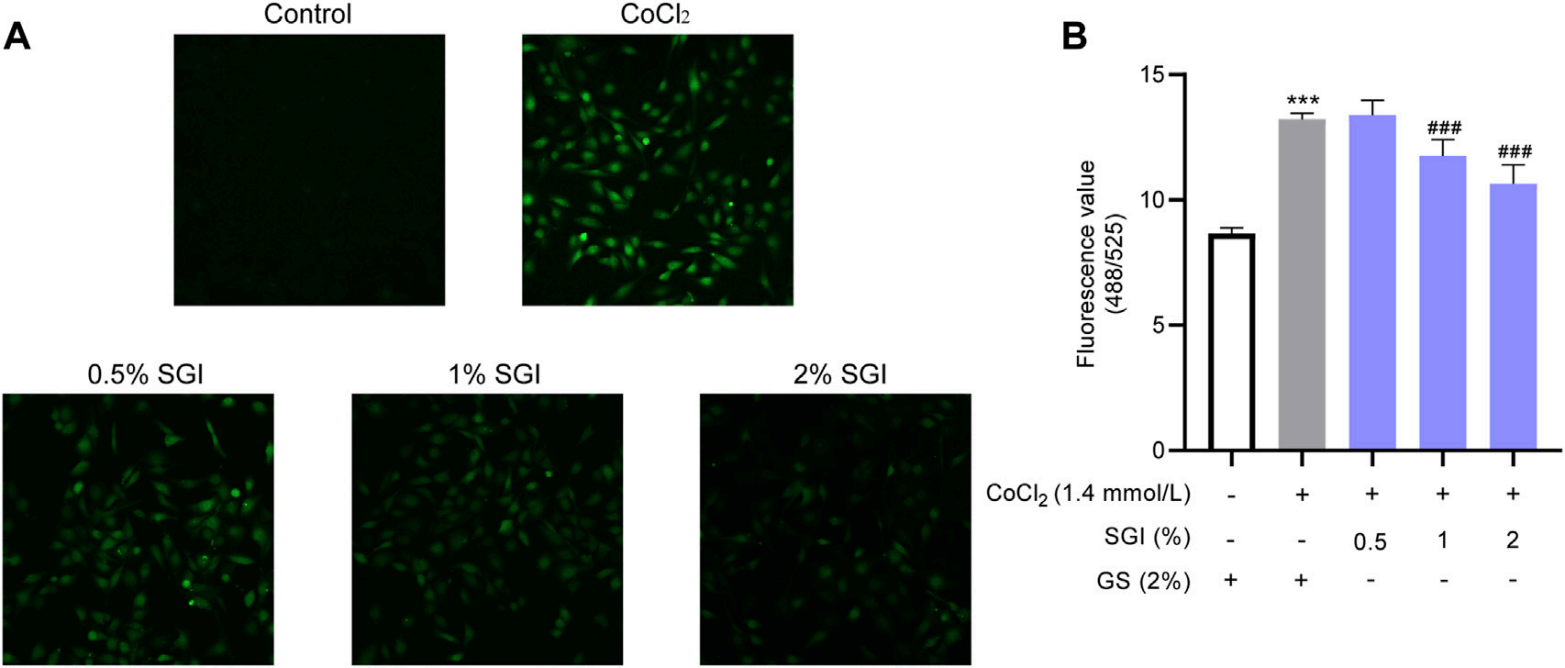
SGI reduces intracellular ROS production. Representative CLSM images of ROS **(A)** and intracellular ROS level **(B)**. ^***^ *p* < .001 vs. the control group; ^###^ *p* < .001 vs. the CoCl_2_ group.

ROS are an important indicators of oxidative damage and the early stage of apoptosis (Wang et al., 2019; Pang et al., 2020). CLSM images showed that, compared with the control group, there was a significant increase in intracellular ROS under the induction of 1.4 mmol/L CoCl_2_. Treatment with SGI (.5%, 1%, and 2%) decreased CoCl_2_-induced ROS production (Figure 5A). Similar results were observed by using a fluorescence microplate reader (SGI at 1% and 2%; *p* < .001, Figure 5B). These findings suggest that SGI could exert antioxidative damage effects in HUVECs.

### 3.5 SGI attenuates CoCl_2_-induced MMP disruption in HUVECs

**FIGURE 6:**
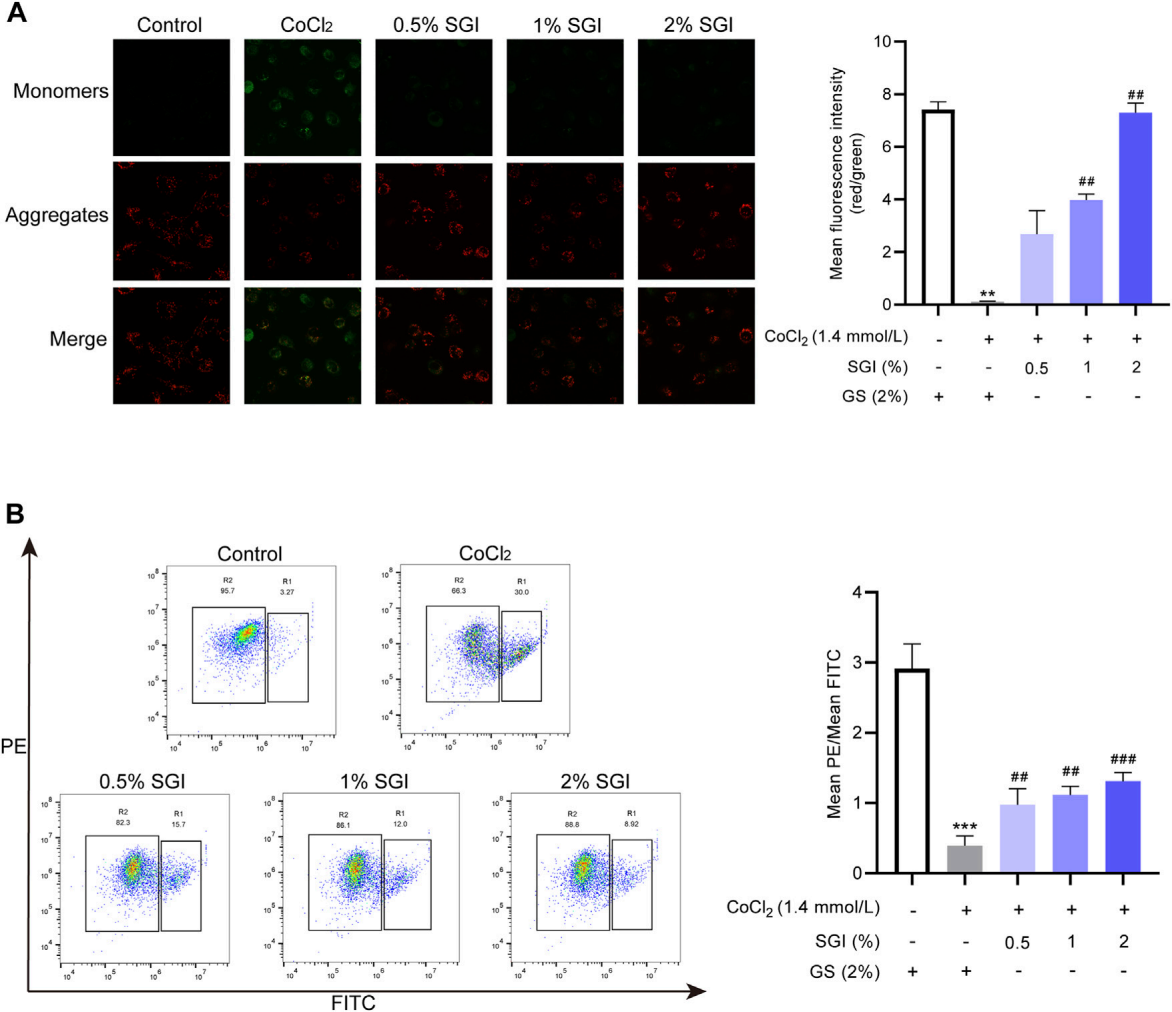
SGI attenuates CoCl_2_-induced MMP disruption in HUVECs. **(A)** Representative CLSM images showing the influence of SGI (.5%, 1%, and 2%) on the MMP and histograms illustrating that ratio of red to green fluorescence. **(B)** HUVECs were analyzed by flow cytometry to quantify the MMP. High MMP is indicated by red fluorescence and low MMP is indicated by green fluorescence. ^**^ *p* < .001, ^***^ *p* < .001 vs. the control group; ^##^ *p* < .01, ^###^ *p* < .001 vs. the CoCl_2_ group.

Intracellular ROS accumulation causes MMP collapse (Cui et al., 2018). CLSM analysis and flow cytometry showed that the MMP of HUVECs was decreased in the CoCl_2_ group in comparison with the control group (*p* < .001), while treatment with SGI (.5%, 1%, and 2%) had the opposite effect (*p* < .01, Figures 6A, B).

### 3.6 SGI decreases cytosolic Ca^2+^ concentrations and G2/M arrest

**FIGURE 7:**
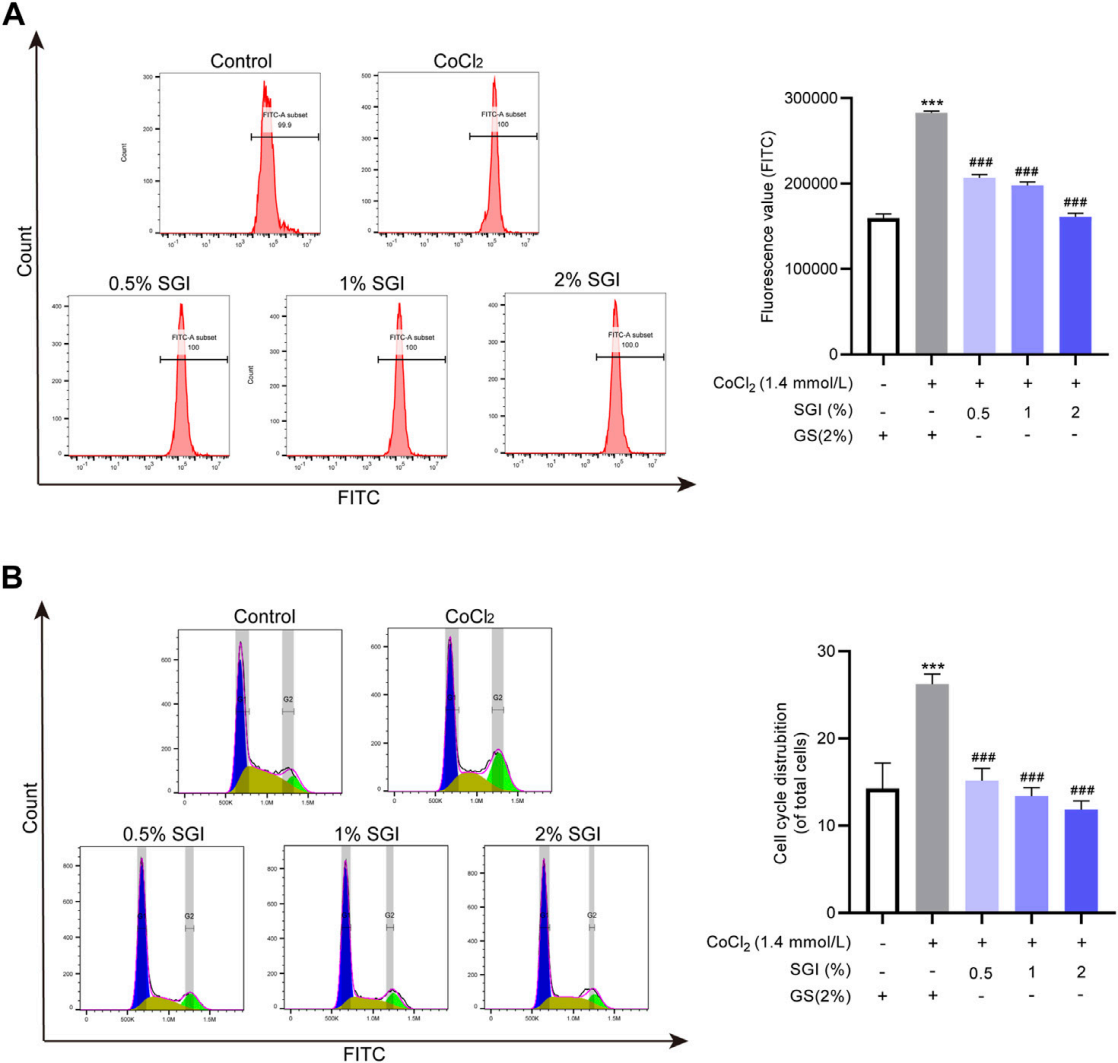
SGI decreases cytosolic Ca^2+^ concentration and G2/M arrest. **(A)** Intracellular Ca^2+^ concentration was assessed by flow cytometry. **(B)** The cell cycle was detected by flow cytometry. ^***^ *p* < .001 vs. the control group; ^###^ *p* < .001 vs. the CoCl_2_ group.

Multiple studies have reported that ROS contributes to intracellular Ca^2+^ overload (Yu et al., 2014; Pang et al., 2020). Consistent with these results, ROS overproduction induced by CoCl_2_ in the current study elevated the level of cytosolic Ca^2+^. However, after treatment with SGI (.5%, 1%, and 2%), the intracellular Ca^2+^ concentration was significantly decreased (*p* < .001, Figure 7A). Moreover, as shown in Figure 7B, CoCl_2_ induced an increase in G2/M arrest in the cell cycle, which was reversed by treatment with SGI (.5%, 1%, and 2%) (*p* < .001). These results suggest that SGI could exert an anti-apoptosis effect by inhibiting the influx Ca^2+^ and the G2/M arrest induced by CoCl_2_.

### 3.7 SGI protects HUVECs from CoCl_2_-induced apoptosis

**FIGURE 8:**
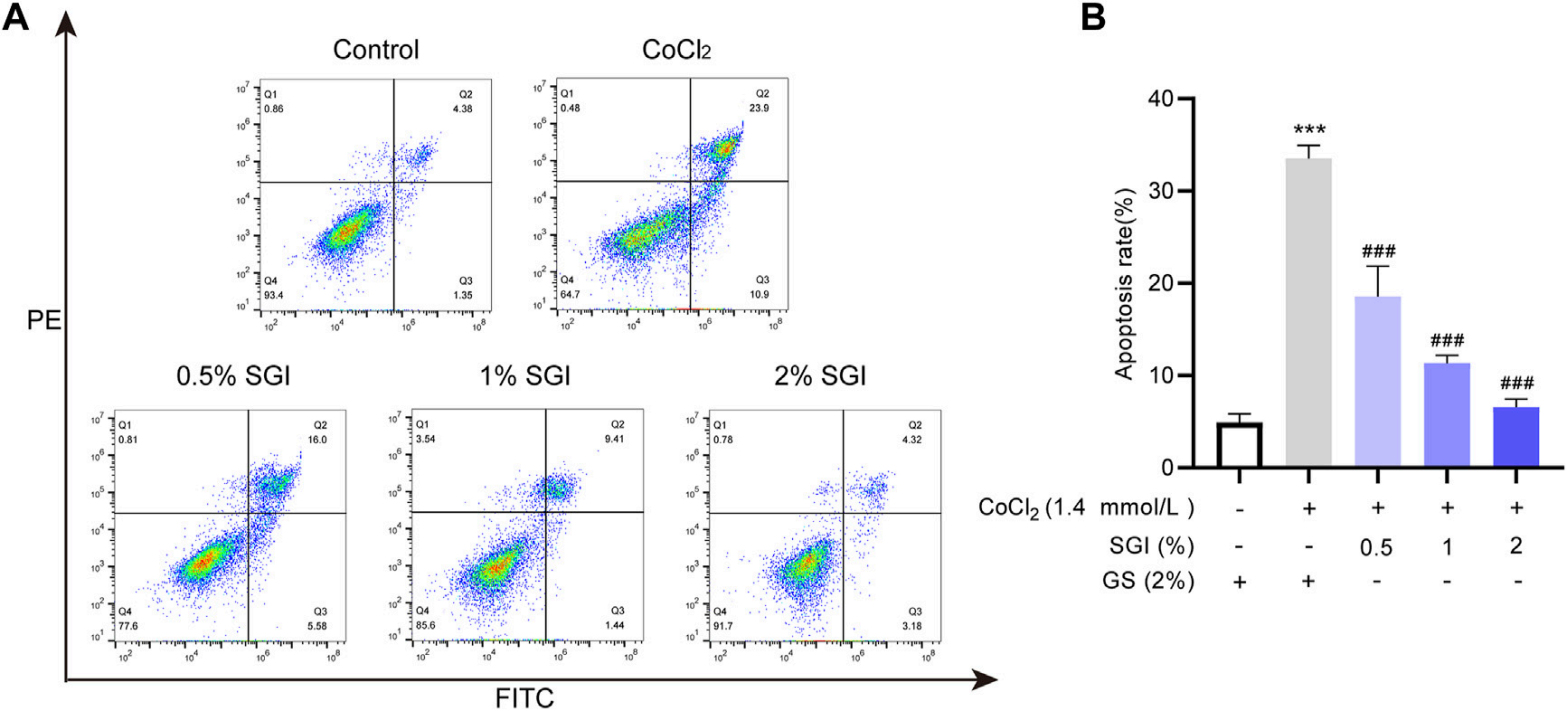
SGI protects HUVECs from CoCl_2_-induced apoptosis. The apoptosis rate was measured by flow cytometry **(A)**, and histograms indicate the proportion of apoptosis **(B)**. ^***^ *p* < .001 vs. the control group; ^###^ *p* < .001 vs. the CoCl_2_ group.

Vascular endothelial cell apoptosis is one of the causes of vascular endothelial dysfunction (Wang et al., 2019). The flow cytometry results showed that the apoptosis rates were significantly increased upon treatment with CoCl_2_ (*p* < .001), while treatment with SGI (.5%, 1%, and 2%) significantly decreased the levels of apoptosis (*p* < .001, Figure 8). These results suggest that SGI could rescue HUVECs from CoCl_2_-induced apoptosis.

### 3.8 Effect of SGI on the expression of apoptosis-associated proteins in CoCl_2_-induced HUVECs

**FIGURE 9:**
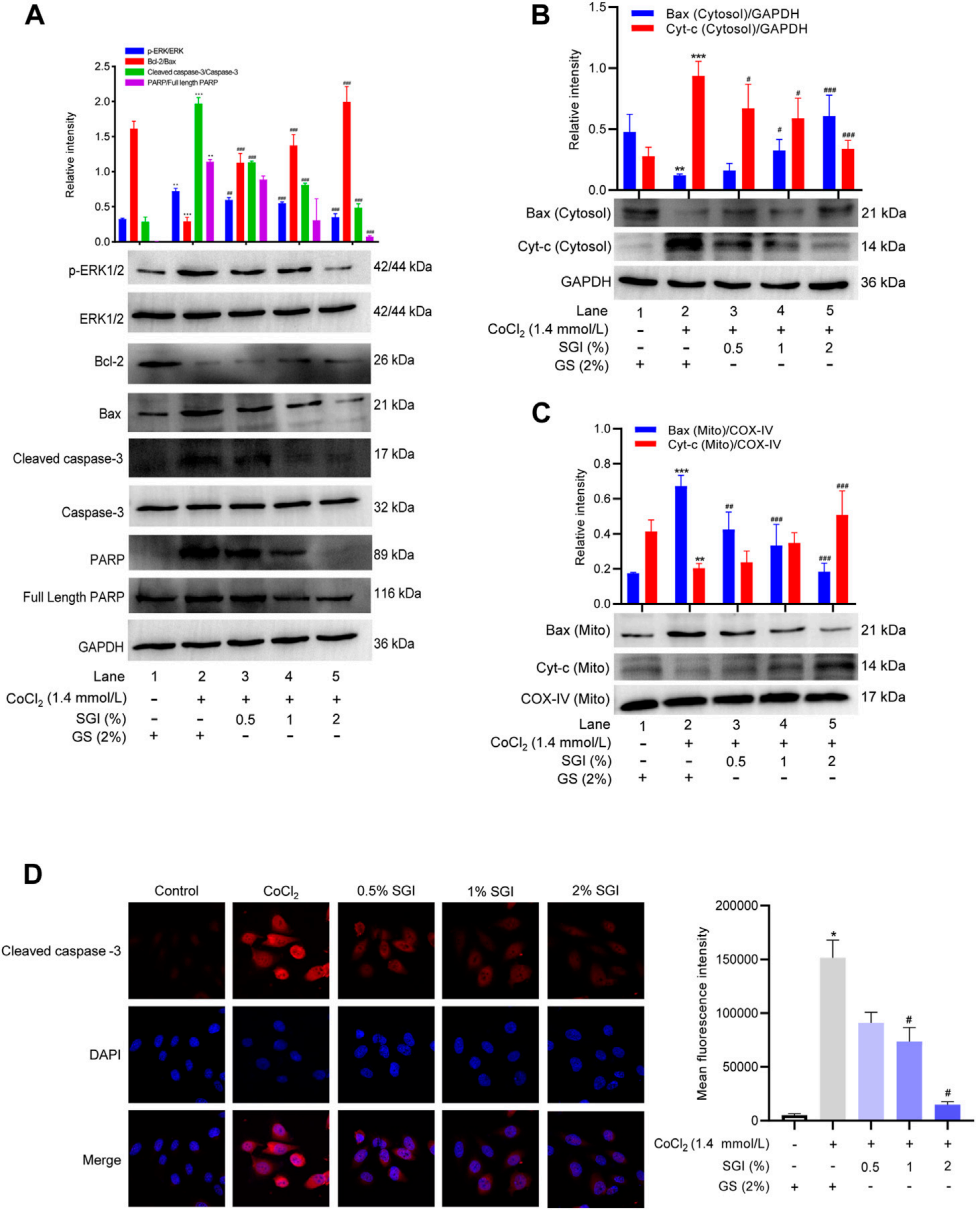
Effects of SGI on CoCl_2_-induced protein expression of HUVECs. **(A)** Protein expression levels of p-ERK/ERK, Bcl-2/Bax, cleaved caspase-3/Caspase-3 and PARP/full-length PARP were measured by western blotting. Histograms represent the statistical analysis of the relative expression level of proteins. GAPDH was used as an endogenous reference. Immunoblot analysis of the expression of Cyt-c and Bax in the cytosol **(B)** and mitochondria **(C)**. **(D)** Immunofluorescence staining of cleaved caspase-3 in HUVECs. ^*^ *p* < .05, ^**^ *p* < .01, ^***^ *p* < .001 vs. the control group; ^#^ *p* < .05, ^##^ *p* < .01, ^###^ *p* < .001 vs. the CoCl_2_ group.

To further elucidate the mechanism of anti-apoptosis effects of SGI, the expression of apoptosis-associated proteins was measured. As shown in Figure 9A, after incubation with CoCl_2_, the phosphorylation level of ERK1/2 was increased (*p* < .01, compare lane 1 to lane 2), the expression of anti-apoptotic protein Bcl-2 was decreased, and the expression of pro-apoptotic protein Bax was increased (*p* < .001, compare lane 1 to lane 2). Treatment with SGI (.5%, 1%, and 2%) increased the Bcl-2/Bax expression ratio (*p* < .001, compare lane 2 to lanes 3, 4, and 5) and decreased the ratio of p-ERK1/2 to ERK1/2 (*p* < .01, compare lane 2 to lanes 3, 4, and 5), compared with the CoCl_2_ group. Additionally, CoCl_2_ induced significant increases in the level of cleaved caspase-3 (*p* < .001, compare lane 1 to lane 2) and PARP (*p* < .01, compare lane 1 to lane 2), which could lead to apoptosis. However, SGI treatment downregulated the ratios of cleaved caspase-3/Caspase-3 (*p* < .001, compare lane 2 to lanes 3, 4, and 5) and PARP/full-length PARP (*p* < .05, compare lane 2 to lanes 3, 4, and 5), and thus protected HUVECs from CoCl_2_-induced oxidative injury and apoptosis. Moreover, similar results in cytoplasmic Cyt-c and mitochondrial Bax after SGI treatment were observed in western blotting (*p* < .001, Figures 9B, C). The immunofluorescence assays also showed downregulation of cleaved caspase-3 in the SGI group (*p* < .001, Figure 9D). These data reveal that SGI protected HUVECs against the apoptosis pathway by maintaining mitochondrial homeostasis and restoring mitochondrial functions.

## 4 Discussion

**FIGURE 10:**
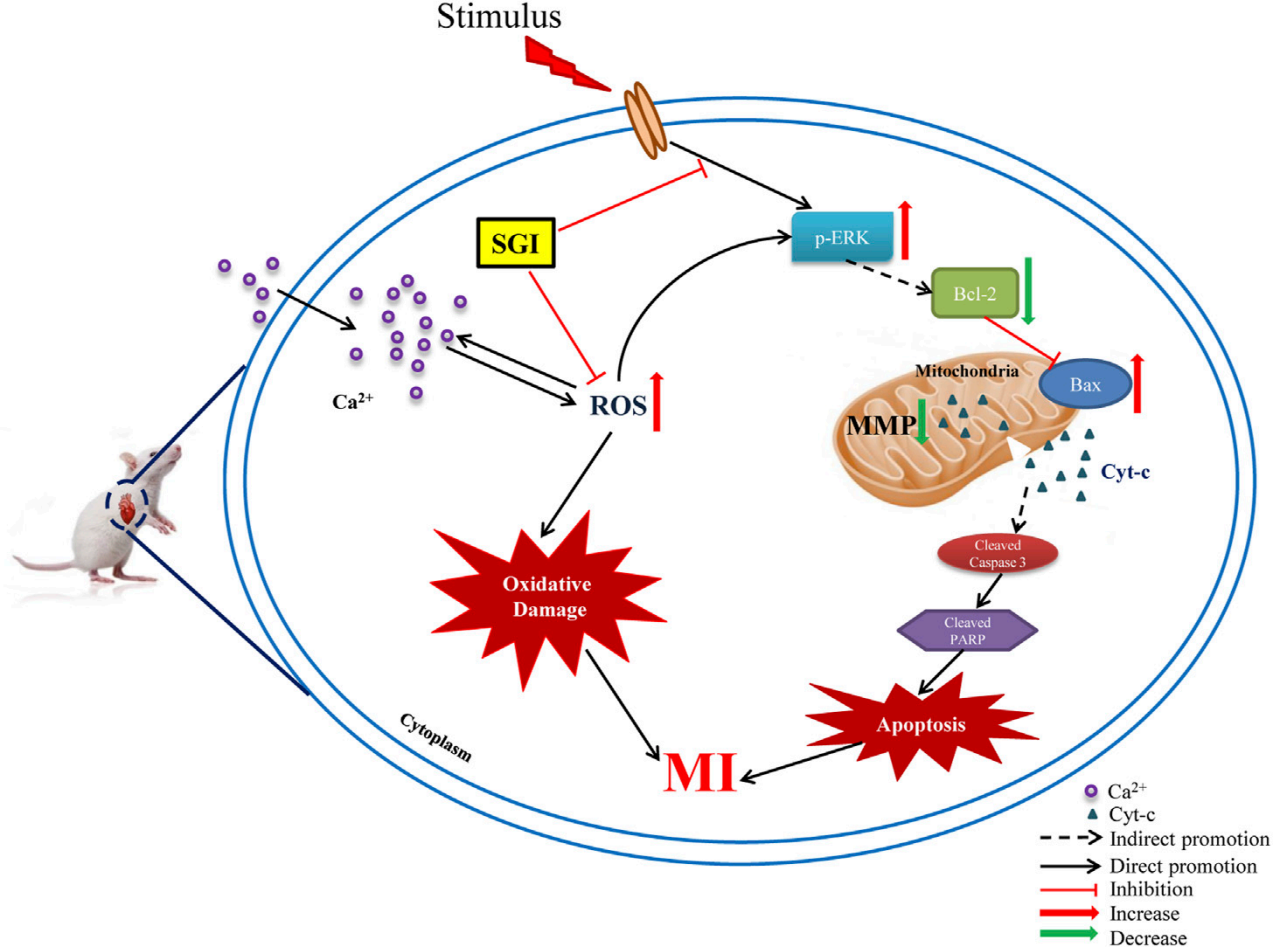
Schematic diagram of the underlying molecular mechanism of SGI against MI. ROS (reactive oxygen species); MI (myocardial ischemia); MMP (mitochondrial membrane potential); p-ERK1/2 (phosphorylation extracellular signal-related kinases1/2); Cyt-c (cytochrome c); PARP (poly-ADP ribose polymerase); Caspase-3 (cysteinyl aspartate specific proteinase 3).

This study showed that SGI treatment attenuated ISO-induced elevation of the ST-segment in ECGs, disorders of echocardiograms, dysfunction of hemodynamic parameters, and overproduction of ROS in heart tissues, and augmented serum concentrations of cTnT and cTnI *in vivo*. Further *in vitro* assays revealed that SGI exerted the effects of antioxidative stress and anti-apoptosis by inhibiting ROS production, MMP collapse, and Ca^2+^ influx, and regulating the expression of apoptosis-related proteins. Therefore, the underlying mechanism of the protective effects of SGI on MI involves inhibition of oxidative stress and apoptosis (Figure 10).

ISO is a recognized drug for inducing MI in animal models through multiple mechanisms, predominantly oxidative stress and Ca^2+^ overload (Xue et al., 2021). Our results showed that ISO can induce ST-segment elevation and a marked increase in ROS, consistent with a previous study (Zhang J. Y. et al., 2022). However, these indicators were significantly decreased in the SGI groups, indicating that SGI could reduce myocardial injury induced by ISO. Moreover, cTnI and cTnT testing has become the standard practice for diagnosis and early exclusion of MI (Apple et al., 2017). In this study, ISO induced an increase in the cardiac damage biomarkers cTnT and cTnI, which was significantly suppressed by SGI treatment. LV systolic and diastolic dysfunction during MI has emerged as an important indicator of cardiac function (Zhang et al., 2016; Fu et al., 2020). In echocardiography, EF(%) and FS(%) values were decreased and LV systolic dysfunction was observed in the MI rats. However, SGI treatment improved LV systolic and diastolic function and changes in hemodynamic parameters. These results demonstrated that SGI improves the recovery of cardiac function after ISO injury.

The vascular endothelium has an important role in the cardiovascular system. The loss of vascular endothelial cell function is a key event in the occurrence and development of vascular diseases (Hou et al., 2015). Vascular endothelial cell dysfunction is predominantly characterized by vascular endothelial cell apoptosis, in which oxidative stress is an important factor (Wang et al., 2019). Multiple studies have demonstrated the importance of protecting vascular endothelial cells against hypoxia-induced injury in CVD (Yang et al., 2018; Wu et al., 2019). CoCl_2_ is a chemical hypoxia modelling reagent that inhibits the catalysis of prolyl hydroxylase in the cell to cause an intracellular hypoxic state, thus creating a hypoxic environment under normoxic conditions (Chen et al., 2018). The apoptosis induced by CoCl_2_ is due to the increased production of free radicals (including ROS) mediated by hypoxia. Increased accumulation of ROS may lead to Ca^2+^ influx. Meanwhile, the increase in intracellular Ca^2+^ concentration also induces additional ROS formation, which leads to mitochondrial dysfunction and further aggravates vascular endothelial cell apoptosis (Yu et al., 2014). Therefore, the balance between the production of ROS and the elimination of excess ROS is essential to maintain the redox state and homeostasis in cells (Liu et al., 2022). Our research has proven that the level of ROS and the concentration of Ca^2+^ increased in CoCl_2_-exposed HUVECs and that SGI treatment protected against hypoxia-induced injury.

There is accumulating evidence that excessive ROS induces apoptosis by phosphorylating and activating the ERK1/2 pathway (Ding Y. et al., 2020). Apoptosis regulation of HUVECs involves phosphorylation of the ERK signaling pathway (Mi et al., 2019). Multiple studies have shown that oxidative stress can lead to the activation of ERK1/2, causing apoptosis (Ding Y. et al., 2020; Huang et al., 2020). In our study, CoCl_2_ increased the phosphorylation level of ERK1/2 and the apoptosis rate of HUVECs. Imbalance between the anti-apoptotic protein Bcl-2 and pro-apoptotic protein Bax (Li J. et al., 2019) can lead to opening of the mitochondrial permeability transition pore (mPTP) and consequent release of cytochrome c (Cyt-c), which then activates apoptosis effector Caspase-3 (Li X. et al., 2019; Pang et al., 2020). Our results demonstrated that pro-apoptotic protein Bax, cytoplasmic Cyt-c, and cleaved caspase-3 were significantly increased in HUVECs induced by CoCl_2_, while expression of the anti-apoptotic protein Bcl-2 protein was decreased, which collectively indicated that the mitochondrial apoptosis pathway was activated. SGI treatment was able to downregulate the expression of Bax, cytoplasmic Cyt-c and cleaved caspase-3 and upregulate the expression of Bcl-2.

Caspase-3 is the most critical apoptosis execution protein that acts on a variety of substrates. Cleaved caspase-3 can further activate PARP (Back et al., 2015). Full-length 116 kDa PARP is cleaved by cleaved caspase-3 into two fragments—24-kDa DNA-binding fragment and 89-kDa catalytic fragment—and thus activated to result in apoptosis (Wei et al., 2012). The G2/M transition in the cell cycle is a major checkpoint that prevents cells from entering mitosis with damaged DNA, thereby maintaining the genomic integrity of offspring (Ding Q. et al., 2020). Our results indicated that SGI could improve the G2/M arrest induced by CoCl_2_ to inhibit apoptosis. SGI could also significantly reduce the cleavage of PARP to inhibit apoptosis.

There is a big gap between the preclinical trials’ effect on antioxidants and the effect of the clinical trial, but the preclinical trials are also important (Qin et al., 2009). As a clinically marketed drug, SGI has shown curative effects in clinical experiments (Sun et al., 2017). Oxidative damage is an important pathophysiological basis for the occurrence and development of MI (Zhang M. et al., 2022). In this study, we found that the effects of SGI against MI occur through inhibitin of oxidative stress and apoptosis pathways. The study provides the scientific basis for the use of SGI in the prevention and treatment of CVD. However, the study does have several limitations. Firstly, the main components of SGI against oxidative stress and apoptosis pathways and the ROS scavenging mechanism of SGI have not yet been elucidated and should be investigated in future studies. Moreover, the antioxidative and anti-apoptotic damage effects of SGI need to be confirmed in subsequent clinical studies.

## 5 Conclusion

SGI is a common medicine used in the clinical treatment of MI in China. The mechanism of SGI may be related to antioxidative stress and anti-apoptosis by reducing ROS production and regulating the intrinsic mitochondrial-mediated apoptosis pathways. The study elaborates on the protective effects of SGI against MI and provides a scientific basis for the action of SGI MI. Furthermore, the study highlights a method and strategy for further investigations on the mechanism(s) of other TCM formulas.

## Data Availability

The datasets presented in this study can be found in online repositories. The names of the repository/repositories and accession number(s) can be found in the article/Supplementary Material.

## Glossary

Bax: Bcl-2-associated X protein
BCA: bicinchoninic acid
Bcl-2: B cell lymphoma-2
BSA: bovine serum albumin
Caspase-3: cysteinyl aspartate specific proteinase 3
CLSM: confocal laser scanning microscope
COX-IV: cytochrome c oxidase IV
cTnI: cardiac troponin I
cTnT: cardiac troponin T
CVD: cardiovascular disease
Cyt-c: cytochrome c
DCFH-DA: 2ʹ,7ʹ-dichlorodihydrofluorescein diacetate
ECG: electrocardiogram
EF: ejection fraction
ERK1/2: extracellular signal-regulated protein kinase1/2
FS: fractional shortening
GAPDH: glyceraldehyde-3-phosphate dehydrogenase
GS: glucose injection
HUVECs: human umbilical vein endothelial cells
ISO: isoproterenol
LV: left ventricular
LVEDD: left ventricular end-systolic diameter
LVESD: left ventricular end-systolic diameter
MI: myocardial ischemia
MMP: mitochondrial membrane potential
mPTP: mitochondrial permeability transition pore
MVE vel: mitral valve E wave peak velocity
NS: normal saline
PARP: poly(ADP-ribose) polymerase
PBS: phosphate buffer saline
p-ERK1/2: phosphorylation extracellular signal-related kinases 1/2
PMSF: phenylmethanesulfonyl fluoride
RIPA: radio immunoprecipitation assay
ROS: reactive oxygen species
SGI: Shenxiong Glucose Injection
SPF: specific pathogen free
TBS-T: tris-buffered saline containingTween 20
TCM: traditional Chinese medicine
