# Supplementary material for: Shenxiong glucose injection inhibits oxidative stress and apoptosis to ameliorate isoproterenol-induced myocardial ischemia in rats and improve the function of HUVECs exposed to CoCl2
Source: Front Pharmacol. 2023 Jan 5;13:931811. doi: 10.3389/fphar.2022.931811 (PMC9849394; doi:10.3389/fphar.2022.931811)
Supplement: Supplementary file 1 [file Table1.DOCX]

Supplementary Material

# Supplementary Figures


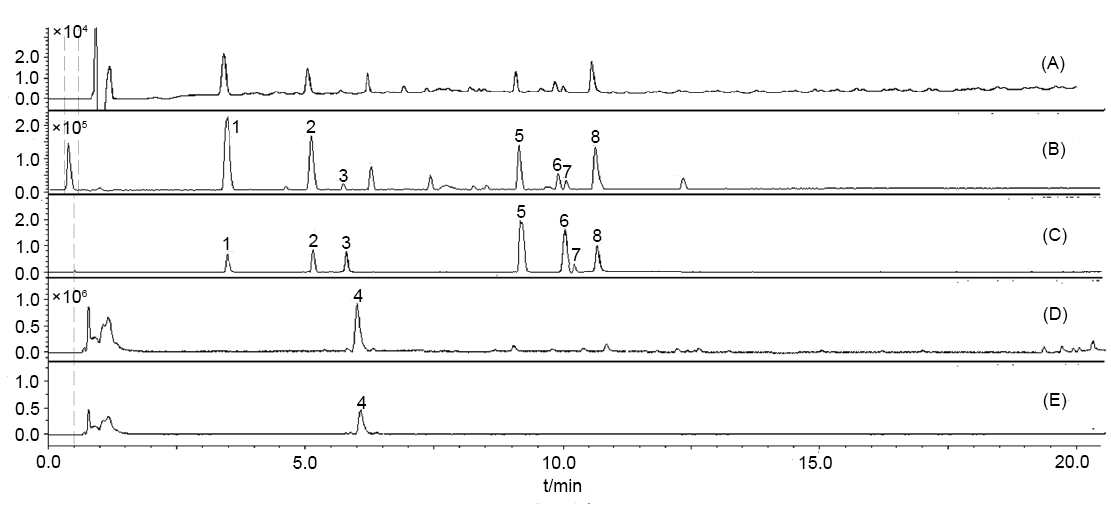


**Supplementary Figure 1.** The chemical profile of SGI. **(A)** Representative UHPLC–DAD chromatogram of SGI. Representative UHPLC–Q–TOF/MS base peak chromatogram of SGI **(B)** and mixture standard solutions **(C)** in negative mode. Representative UHPLC–Q–TOF/MS base peak chromatogram of SGI **(D)** and reference solution (peak 4, ligustrazine hydrochloride) **(E)** in positive mode. Adapted with permission from reference (Zheng, 2015). Peak 1, danshensu; peak 2, protocatechualdehyde; peak 3, caffeic acid; peak 5, salvianolic acid D; peak 6, rosmarinic acid; peak 7, salvianolic acid A; and peak 8, salvianolic acid B.
